# Ocean Sensitivity to Periodic and Constant Volcanism

**DOI:** 10.1038/s41598-019-57027-0

**Published:** 2020-01-15

**Authors:** Muhammad Mubashar Dogar, Tomonori Sato, Fei Liu

**Affiliations:** 1 Global Change Impact Studies Centre, Ministry of Climate Change, Islamabad, Pakistan; 2 JSPS Fellow, Hokkaido University, Sapporo, Japan; 3 Faculty of Environmental Earth Science, Hokkaido University, Sapporo, Japan; 4 Earth System Modeling Center and Climate Dynamics Research Center, Nanjing University of Information Science and Technology, Nanjing, China

**Keywords:** Atmospheric dynamics, Climate change, Climate and Earth system modelling

## Abstract

It is strongly believed that the explosive eruptions produce negative radiative forcing that causes long-term perturbations in the ocean. Moreover, it is anticipated that a sporadic strong cooling should initiate more vigorous vertical mixing of the upper ocean, and therefore cools the ocean more effectively than a uniform radiative forcing. However, the long-term simulations show that on average the ocean heat content responses to periodic and constant forcings are comparable. To better understand this controversy and to better quantify the post-eruption oceanic response, we conducted two sets of parallel simulations, the first with a uniform/constant volcanic forcing and the second one with a periodic volcanic forcing of magnitude 1×, 5×, 10× and 30× of Pinatubo size eruption using Geophysical Fluid Dynamics Laboratory’s coupled model, CM2.1. We systematically compared the effect of periodic volcanic forcing with an equivalent time-average volcanic cooling. Our results reveal that on average, volcanic-induced perturbations in Ocean Heat Content (OHC), and sea-level rise (SLR) following uniform and periodic eruptions are almost identical. It further emphasizes that the strength of ocean heat uptake at different ocean depths is mainly driven by the strength of the Atlantic Meridional Overturning Circulation (AMOC). These findings are important for ocean initialization in long-term climate studies, and geoengineering applications. It would help to unfold uncertainties related to ocean relaxation process, heat storage, and redistribution.

## Introduction

Volcanic eruptions constitute an important external forcing of the climate system^1–3^. Volcanic induced climatic impacts have been largely analyzed in terms of their atmospheric thermal and dynamical anomalies^4–11^. Large explosive volcanic eruptions are generally considered as a fairly good analogue for stratospheric aerosol injection in climate engineering experiments as they produce global cooling that counteracts global warming^12–16^. This is another reason why they received much attention in the past few years. However, fewer studies have focused on their effect on the oceans probably because of the lack of reliable reconstructions and instrumental records^17–19^. Therefore, the effects of volcanic eruptions over oceans are not completely known^19,20^. Hence, for a better understanding of the post-eruption role of oceans in the climate system, quantification of oceanic ability to store and transport heat and associated changes in sea-level rise and ocean circulation is necessary. Moreover, the analysis of ocean sensitivity following explosive volcanism is vital, as it could help to overcome possible uncertainties in ocean heat content and sea level changes and ocean’s relaxation process^14,21,22^.

It was long suspected that very strong explosive volcanic events could cause long-lasting climate perturbations that are capable of even triggering an ice age^23,24^. This depends strongly on how effectively volcanic forcing could cool the ocean. Due to nonlinearities associated with the aerosol plume formation and climate processes, climate response does not scale with respect to the magnitude of a volcanic eruption. Even very strong sporadic volcanic events affect mostly the upper ocean and equilibrate in about a decade^14,18,25^. A sporadic volcanic eruption produces sudden cooling impact over the ocean surface, and as the eruption event is over, the upper ocean begins to absorb more solar radiation, interacts with the quickly recovering atmosphere, and warms. However, the volcanic cooling signal penetrates down towards the deep ocean^25^.

In earlier studies^14,26,27^ it is argued that the ocean response could be different for periodic/sporadic and equivalent time-mean forcing due to different vertical mixing in the ocean. In these studies it was believed that the sporadic strong volcanic cooling intensifies vertical mixing processes in ocean due to increased turbulent diffusion, seasonal convection, and overturning^14,27,28^. However, the long-term simulations show that on average the ocean heat content and sea-level rise responses to periodic and constant forcings are almost identical^29,30^.

To better understand this controversy, in this paper, we will study how the impact of a periodic volcanic forcing accumulates in the deep ocean and if it could cause a stronger effect than an “energetically equivalent” uniform volcanic impact. This could help to better understand if periodic events, like Siberian or Deccan Traps, have more potential of causing strong climate perturbations than sporadic super-eruptions of varying strength. For this purpose, we designed several sensitivity experiments with varying strength and frequency of volcanic aerosol forcing. We used the CM2.1, the GFDL coupled atmosphere-ocean climate model to conduct cyclic simulations with 1×, 5× 10× and 30× of Pinatubo forcing, simulated for 10, 50, 100 and 300 years repeating cycles respectively and energetically equivalent time-mean volcanic aerosols simulations (see section 2, Model and Experimental Setup, for details). These idealized periodic and constant volcanic forcing experiments will help us to better understand the role of oceans in heat adjustment and redistribution process that in turn could help to better quantify the uncertainties in ocean heat content and sea-level changes^21,22^. In this paper, we focus on the following questions.

1. How effectively periodic and energetically equivalent constant volcanic eruptions cool the upper and deep ocean?
2. What is the response of OHC, SLR and AMOC to the periodic and constant volcanism of varying strength (i.e., 1×, 5× 10× and 30× of Pinatubo strength)?

The rest of the paper is organized as follows. Section 2 briefs the experimental setup used for the study of the ocean response to volcanic forcing. In section 3, we presented results and discussion in which the response of OHC, SLR, AMOC and volcanic-induced Atlantic Ocean temperature changes are discussed. In the last section, the summary and conclusion of this study are given.

## Model and Experimental Setup

The Geophysical Fluid Dynamics Laboratory (GFDL)’s coupled climate model version 2.1 (CM2.1) is employed here^31^ to assess the ocean’s response to explosive volcanism. The GFDL-CM2.1 model is composed of four component models viz. atmosphere, ocean, land and sea ice. This model has been widely used for the study of the volcanic signals in the oceans and for studying the impact of volcanic forcing on ENSO teleconnection^14,32^. Detailed documentation of CM2.1 model and relevant references can be seen at (http://nomads.gfdl.noaa.gov/CM2.X/references/). The initial conditions for the volcanic ensemble simulations conducted in this paper are taken from the CM2.1 based long control runs that were calculated during the preparation of the Intergovernmental Panel on Climate Change Fourth Assessment Report (IPCC AR4) project. This long control run was conducted for 4000 years, to bring the deep ocean close to the equilibrium state, with all the other climate forcings held fixed at 1860 level.

To study ocean’s response to explosive volcanism of different magnitude, we selected Mt. Pinatubo, June 1991 eruption as a test-bed and increased its magnitude to 5×, 10× and 30× of Pinatubo size. The Mt. Pinatubo eruption occurred in the satellite era and is the best observed explosive eruption event^33–38^. It allows better testing of the modeling framework^14^. During this eruption, about 17 to 20 Teragrams (Tg) of SO2 were injected into the lower stratosphere and subsequently converted into sulfate aerosols^14^. The radiative forcing for this event is best constrained by instrumental observations from the space-borne platforms^39,40^. Table 1 summarizes the set of control and perturbed volcanic forcing experiments considered in this paper.

To filter out the effects of random noise in the output volcanic-induced climate signal, we produced three realizations and examined the results as an average of these three realizations. The control and perturbed runs use the same initial condition, however, the only difference among them is that the perturbed simulations are forced by the volcanic forcing whereas control simulations do not contain this volcanic input. The volcanic aerosol forcing data used in the model is calculated interactively within the model based on the aerosol optical characteristics of Pinatubo eruption as discussed by^14,39^. The perturbed volcanic experiments are conducted using one time (1×), five times (5×), ten times (10×) and thirty times (30×) of Pinatubo forcing each for periodic volcanic aerosol forcing and energetically equivalent constant (time-mean) volcanic aerosol forcing. In the case of periodic simulations with 1×, 5×, 10× and 30× Pinatubo forcing, the repeating cycles are, respectively, 10, 50, 100 and 300 years. Both the periodic (1×, 5×, 10× and 30×) and equivalent time-mean volcanic aerosol experiments are run for 300 years time period (see Table 1). We produced three-ensemble members both for the control and perturbed (periodic and time-mean) cases. The control and individual perturbed ensemble simulations share the same initial condition and in total three different initial conditions are used for the three ensemble simulations and the results are presented as an average response of these three ensemble members. The results are shown in the form of anomalies. To filter any possible drift in the responses, these anomalies are computed by subtracting corresponding control runs from the perturbed runs, which has the same drift, as was done in previous studies^14,32,41^.

**Table 1:** Experimental setup used for the study of ocean response to periodic and time-mean volcanic forcing.

CM2.1 Experiment Details					
Experiment Name	SW Forcing	LW Forcing	Periodic Cycle	Ensembles	Duration (years)
Control	None	None	None	3	300
Uniform (Time-mean)	Uniform	Uniform	None	3	300
1× volcano forcing	1× Pinatubo	1× Pinatubo	10 Years	3	300
5× volcano forcing	5× Pinatubo	5× Pinatubo	50 Years	3	300
10× volcano forcing	10× Pinatubo	10× Pinatubo	100 Years	3	300
30× volcano forcing	30× Pinatubo	30× Pinatubo	300 Years	3	300

For comparison, the volcanic aerosol forcing for time-mean case over 10 years period is made energetically equivalent to 1× Pinatubo per decade. A similar setup is done for the simulations of 5×, 10× and 30× periodic simulations and their energetically equivalent uniform simulations. Further details regarding the volcanic aerosol forcing input data and the experimental setup is given below.

To simulate the volcanic perturbation, we prescribe the Pinatubo aerosols’ optical properties according to Stenchikov *et al*.^14^ using aerosol optical depth (AOD) from Sato *et al*.^42^. This volcanic data set provides zonally averaged monthly mean spectral dependent aerosol extinction, single scattering albedo, and an asymmetry parameter which are required to conduct radiative transfer simulations within CM2.1. The Pinatubo size forcing is applied in June 1991. The control and perturbed runs first diverge in mid-May 1991 because the monthly mean aerosol characteristics are interpolated between the months.

**Figure 1:**
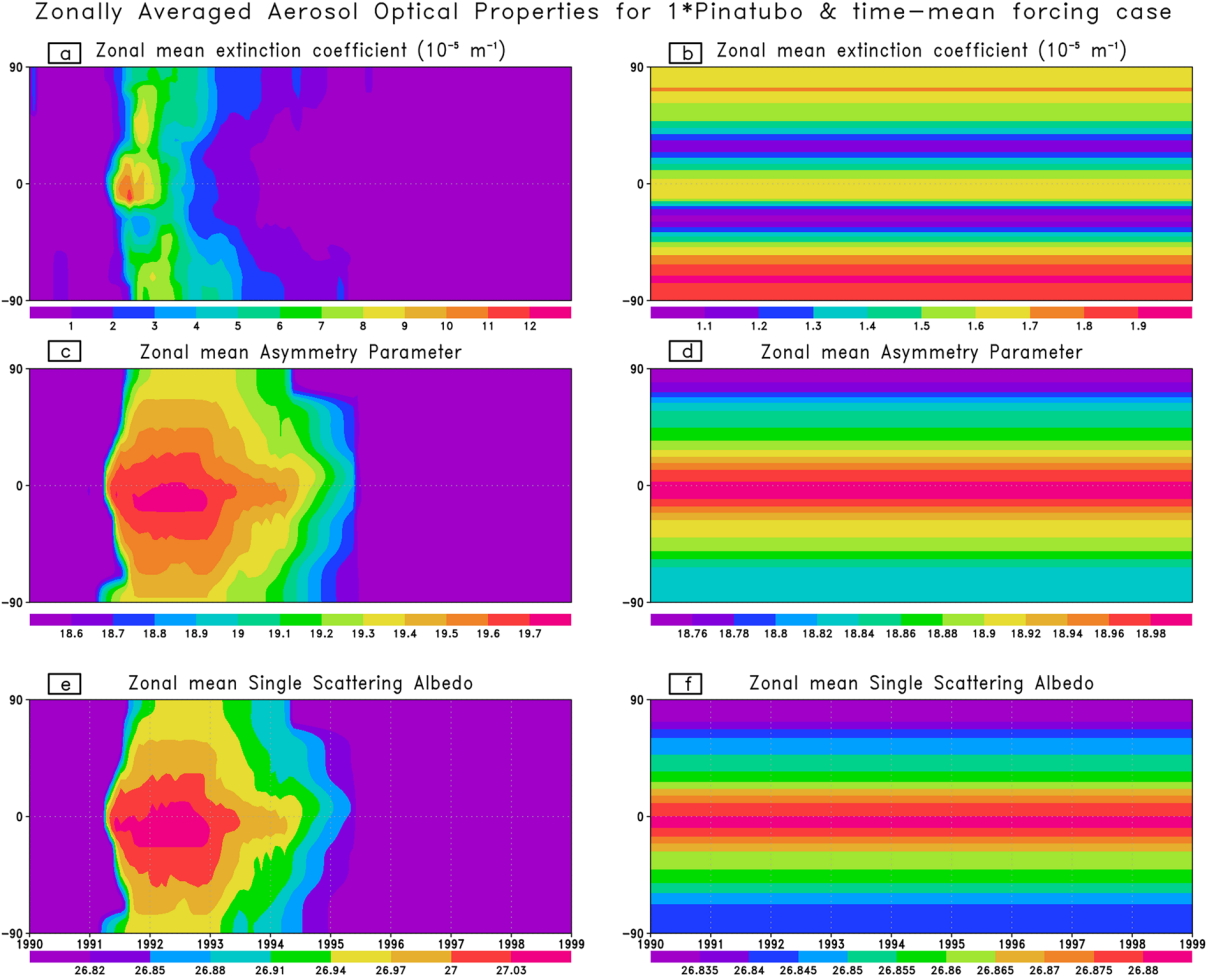
Vertically summed zonal mean aerosol extinction coefficient, asymmetry parameter and single scattering albedo for 1× Pinatubo/10a (left column) and time-mean volcanic forcing (right column), displayed by averaging over the model spectral bands in short wave range.

The spatial and temporal distribution of the aerosol optical properties for 1× Pinatubo/10a and energetically equivalent (i.e., time-mean) volcanic forcing used in the model is shown in Fig. 1. These aerosol properties are displayed as vertically summed zonal mean monthly values of extinction coefficient, asymmetry parameter, and single scattering albedo for 1× Pinatubo/10a and energetically equivalent time-mean volcanic experiment. These parameters are displayed in Fig. 1 by averaging over the entire range of model short wave spectral bands. For 5×, 10× and 30× Pinatubo size volcanic experiments, these parameters (i.e., extinction coefficient, asymmetry parameter and single scattering albedo) were scaled to 5×, 10×, and 30× of Pinatubo strength using AOD from Sato *et al*. [1993]. For periodic case, the aerosol radiative forcing develops during the time of eruption and relaxes with an e-folding time of 1–2 years (Fig. 1, left column). For energetically equivalent volcanic case, the aerosol radiative forcing is uniformly distributed over the entire simulation period (Fig. 1, right column) so that the total aerosol forcing is the same to the periodic case.

We conducted three ensemble integrations of control (without volcanic aerosols) and perturbed experiments (periodic volcano forcing of 1×, 5×, 10×, 30× of Pinatubo strength and corresponding time-mean volcano forcing). The ensemble of three different atmospheric initial conditions is intended to capture the intrinsic variability of the climate system. To increase the signal-to-noise ratio, we present our results using ensemble over the volcano runs minus the ensemble mean over the corresponding segments of the control runs.

## Results and Discussion

### Ocean heat content and sea-level rise responses

Previous modeling experiments on volcanic forcing suggest that historical volcanic eruptions tend to offset a large fraction of the ocean heat content (OHC) and global mean sea-level (GMSL) increase caused by anthropogenic forcing in the 20th century^25,41^. Moreover, changes in the energy budget of the climate system are predominantly revealed in ocean temperatures and the associated contribution of the ocean thermal expansion that result towards an increase of the ocean heat content and sea-level rise^43,44^.

**Figure 2:**
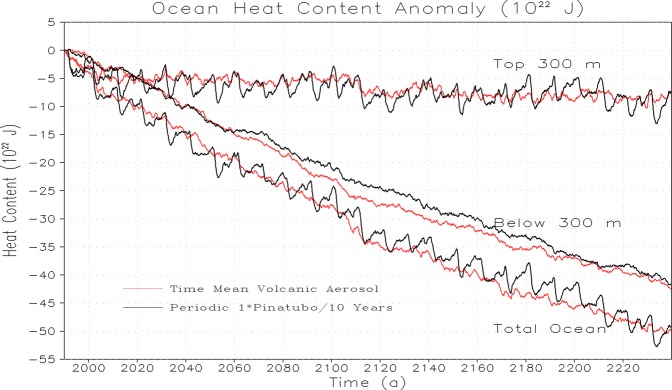
Temporal evolution of change in Ocean Heat Content (OHC) relative to corresponding control for periodic 1× Pinatubo and time-mean volcanic aerosol simulation.

**Figure 3:**
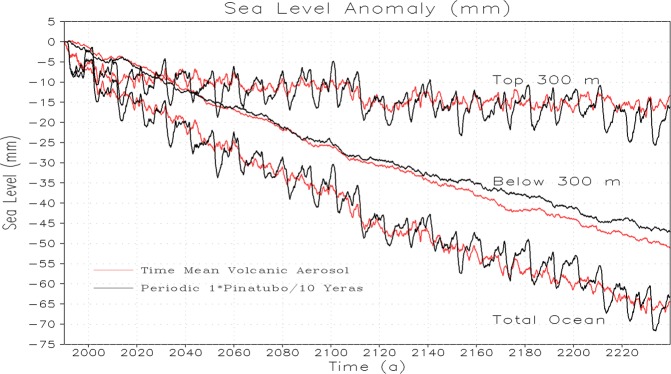
Temporal evolution of change in sea-level rise (SLR) relative to corresponding control for the periodic 1× Pinatubo and time-mean volcanic aerosol simulation.

In order to better understand the oceanic responses to periodic and time-mean volcanic forcings, we analyzed the ocean heat content and mean sea level responses following the volcanic eruptions. We find that the average ocean heat content and global mean sea level decrease are same both for the periodic volcanic forcing of Pinatubo size and energetically equivalent time-mean volcanic forcing for upper 300 m, below 300 m, and total ocean depth (Figs. 2 and 3). The upper ocean shows sudden cooling following the strong periodic eruption, whereas, the response of the upper ocean to energetically equivalent constant volcanism is relatively small in the beginning, however, both the parallel experiments (i.e., 1× periodic and time-mean volcanic forcing of Pinatubo size) reach nearly the same cooling level at the end of the simulation period. The OHC decrease and associated sea-level rise response in the upper 300 m layers indicate that the upper ocean is getting stable and tries to recover and relax back following each eruption because it interacts (through air-sea interaction) and exchanges heat with the associated upper atmosphere.

**Figure 4:**
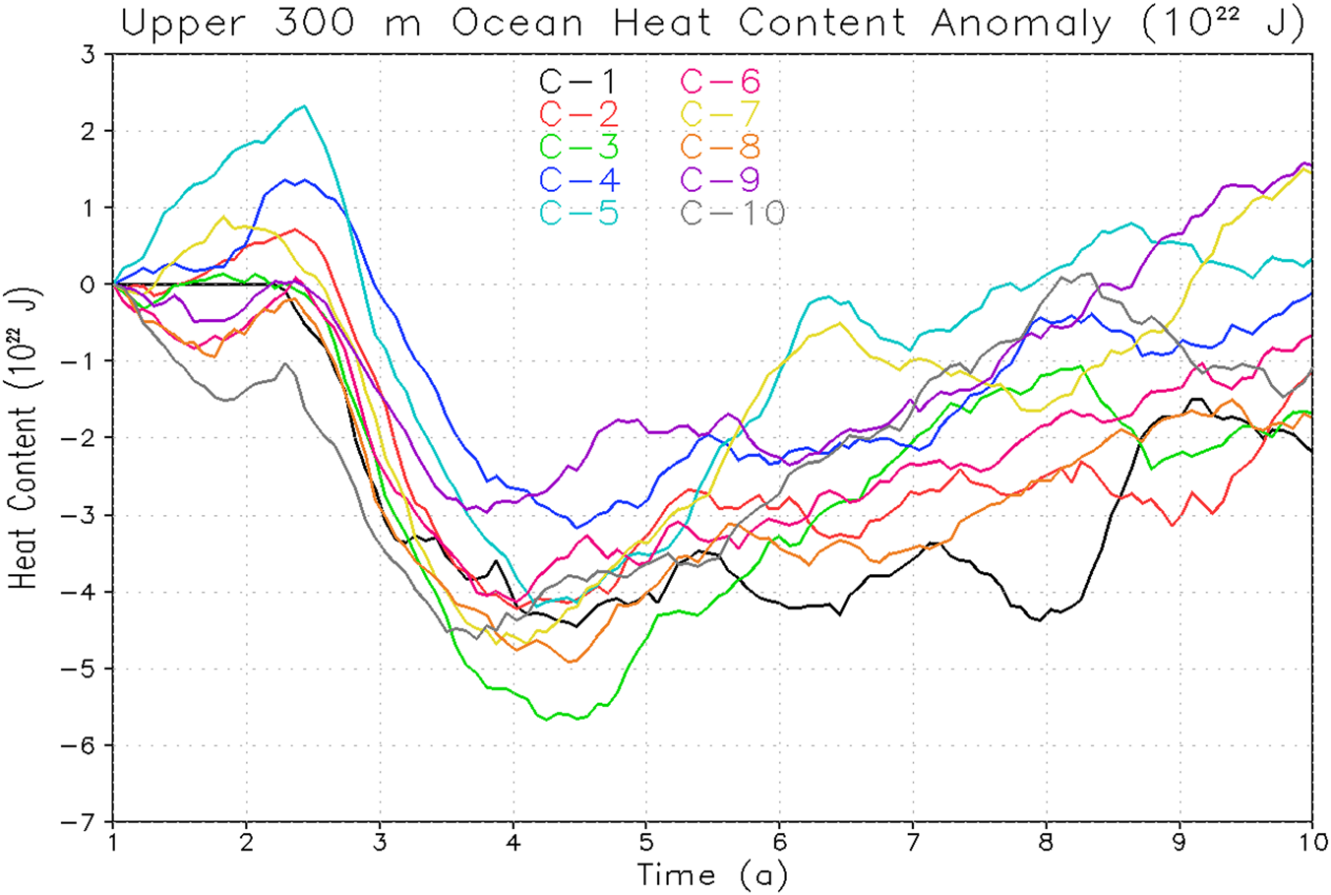
Temporal evolution of change in Ocean Heat Content (OHC) relative to control for periodic 1× Pinatubo first 10 cycles with reference to zero starting point for upper 300 m. The legends C-1 to C-10 represents to cycle 1 to cycle 10 respectively.

**Figure 5:**
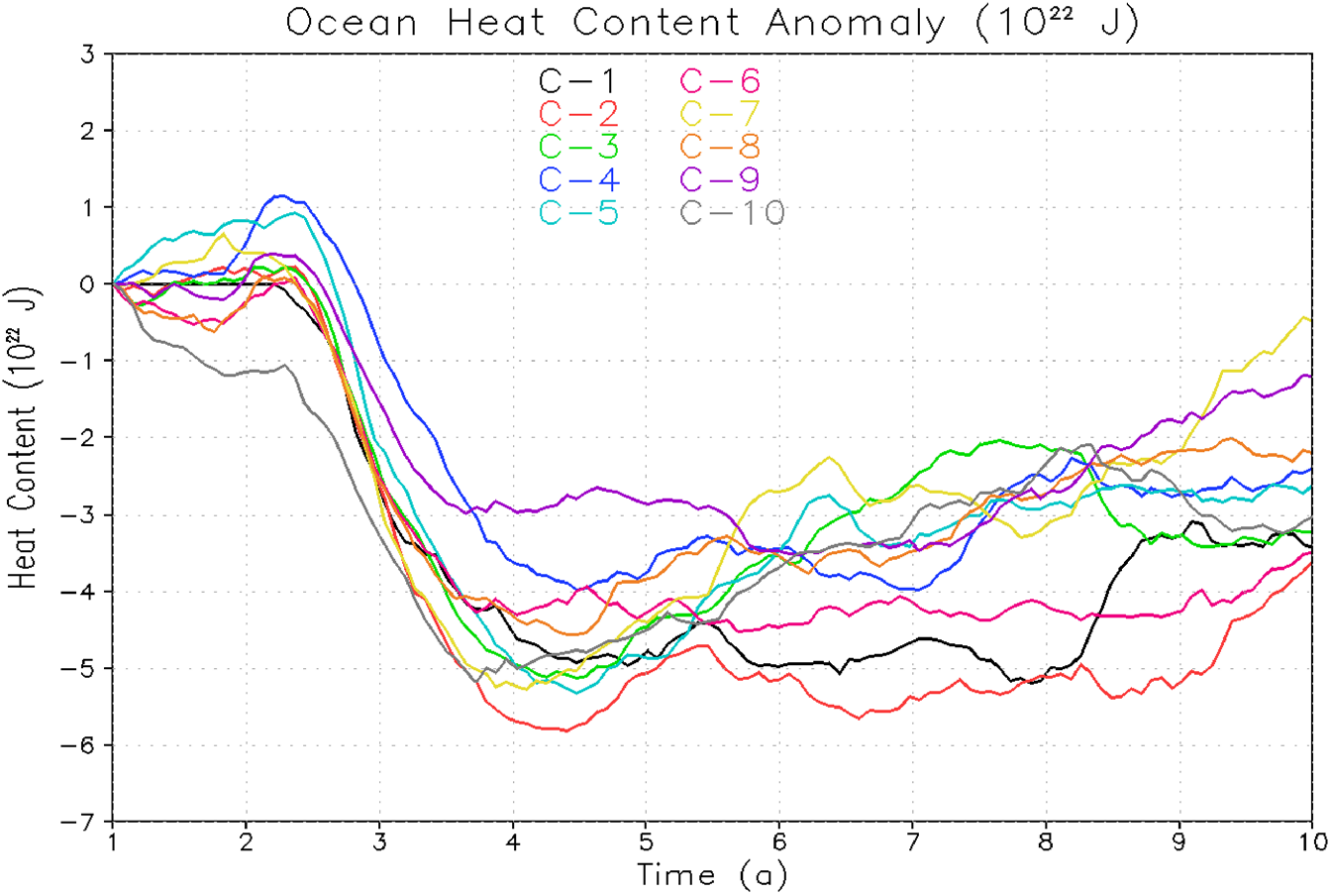
Temporal evolution of change in Ocean Heat Content (OHC) relative to control for periodic 1× Pinatubo forcing first 10 cycles with reference to zero starting point for total ocean depth.

It is believed that the ocean relaxation process after climate impact of a single eruption is different than the effect of the cyclic eruptions of same cumulative magnitude^26,27^. These studies looked at the OHC and temperature response for the entire ocean. However, volcanic signals need to be analyzed separately in the upper and deep ocean as these could respond differently resulting in significantly different relaxation process for the upper and lower ocean. Hence, to better understand the response of upper, and deep ocean to periodic eruptions and their post-eruption relaxation process, we analyzed OHC responses (anomalies are computed with respect to corresponding control simulation) for the first 10 cycles of 1× Pinatubo/decade case for upper 300 m and total ocean depth and plotted them with reference to their starting reference value (Figs. 4 and 5). The maximum OHC decrease both for top 300 m depth (Fig. 4) and entire ocean depth (Fig. 5) after each cycle is almost identical and the upper ocean is playing the main role in the relaxation process as it interacts faster with the upper atmosphere. However, the lower ocean (below 300 m) accumulates the cooling signal and therefore its relaxation response is relatively slower. It happens when a significant portion of the ocean cold anomaly after the volcanic eruption penetrates down and accumulates into the deep ocean layers; the pace of the vertical energy exchange reduces and relaxation slows down.

**Figure 6:**
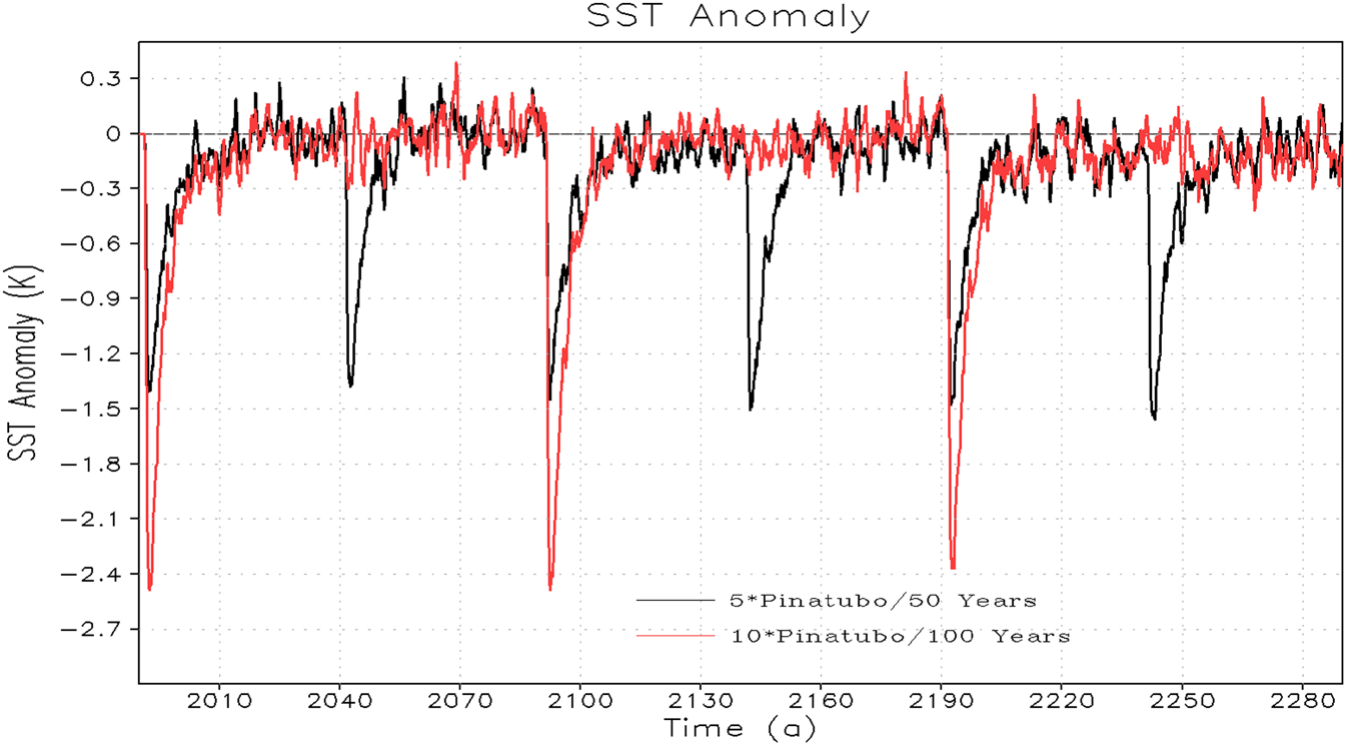
Temporal evolution of change in Sea Surface Temperature (SST) relative to control for periodic Pinatubo (5× and 10× cases) first 300 years.

**Figure 7:**
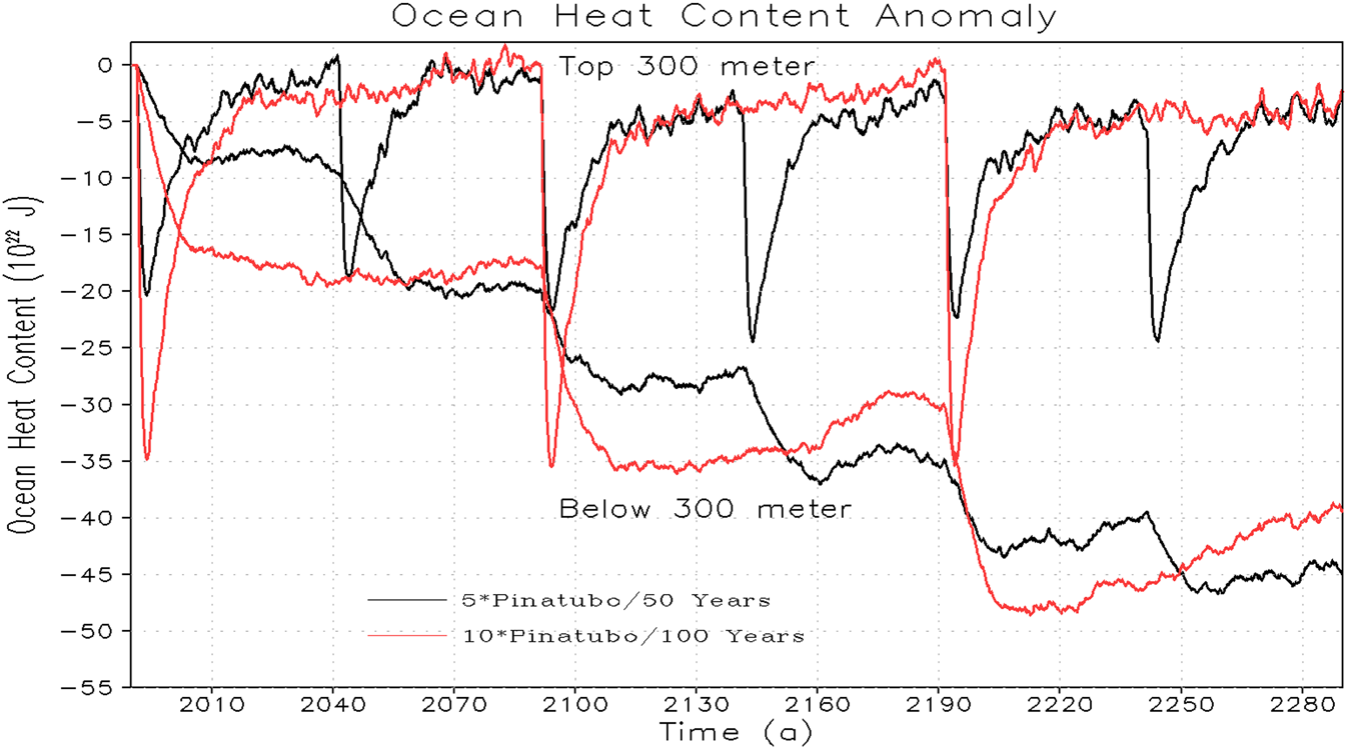
Temporal evolution of change in Ocean Heat Content (OHC) relative to control for periodic Pinatubo (5× and 10× cases) for the first 300 years.

The sea surface temperature (SST) and OHC responses (both for the top 300 m and the deep ocean) to periodic eruptions with increased strength up to 5× and 10× of Pinatubo forcing and energetically equivalent time-mean volcanic forcing are shown in Figs. 6 and 7 respectively. The SST anomaly takes a sudden step down following each periodic volcanic eruption and then recovers back to the background level due to the heat exchange process between the ocean surface and the associated upper atmosphere. It follows Newton’s law of cooling and heating, which states that the rate of heat loss or gain of a body is directly proportional to the difference in the temperatures between the body and its surroundings. The upward transfer of heat by the upper ocean surface is much stronger following each periodic eruption event^26^ resulting in a fast recovery of SST. The post-eruption reduction (i.e., maximum decrease) of the globally averaged SST anomaly is doubled with the doubling of the magnitude (5× to 10× Pinatubo) of the volcanic forcing (Fig. 6). The OHC response for the upper 300 m is largely consistent with SST response, however, as expected the deep ocean accumulates the cooling signal and therefore OHC anomaly for the lower ocean decreases continuously following each eruption cycle. The signature of the post-eruption cooling signal is long lasting in the deep ocean layers compared to the upper ocean layers. The recovery of the OHC in the deep ocean is relatively slower probably because the upward heat transfer from ocean interior layers towards the upper ocean surface and the associated atmosphere is relatively slower. It is interesting to note that the response of OHC decrease in the deep ocean (below 300 m) is quantitatively comparable for 1×, 5× and 10× cases both in periodic and constant (time-mean) volcanic forcing scenarios (Figs. 2 and 7) indicating that the deep ocean integrates cooling signal for massive super-eruptions^29^.

We noticed that the SST and the upper ocean (top 300 m) almost return to their unperturbed state at the end of each cycle (obvious in the first one to two cycles) of periodic eruptions (Figs. 6 and 7). It happens due to strong vertical mixing and energy exchange between the atmosphere and the upper ocean following strong volcanism. This analysis suggests that for the smaller eruptions, upper ocean require relatively less relaxation time whereas for stronger massive eruptions, it requires relatively more time to relax, which in our case is approximately 10 years for a Pinatubo size eruption and almost 50/100 years for 5×/10× Pinatubo size eruptions. This relaxation criterion seems effective for initial cycles of each periodic volcanism of different magnitudes. Nevertheless, when a significant portion of an ocean cold anomaly penetrates to depth, the pace of the vertical energy exchange decreases and relaxation slows down and therefore the entire relaxation process might take longer time that varies between decades to centuries^14^. It happens presumably because in the first few cycles of periodic volcanism, the relaxation is mostly driven by the direct ocean-atmosphere interaction. In the subsequent cycles, the relaxation in part is driven by the processes of ocean vertical mixing which include seasonal convection, Ekman pumping, mixing in subtropical gyres, upwelling/downwelling, and overturning^14^. Our results are consistent with the findings shown in previous studies showing the eruption of Pinatubo in 1991 that led to rapid reductions in OHC and GMSL, with recovery process taking up to a decade^18,25,41^. The SST and OHC response for periodic and time-mean forcing scenario is identical, and it happens because upward heat exchange for time-dependent periodic case varies between maximum and minimum (it is stronger just after the eruption and then decays with time), whereas for the case of time-mean volcanic forcing the upward heat exchange is continuous^26^, making on average equivalent heat exchange for the periodic and time-mean volcanic scenarios.

**Figure 8:**
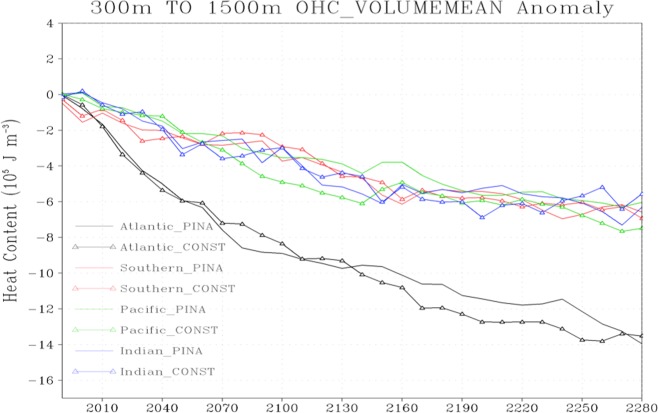
Temporal evolution of change in volume-mean Ocean Heat Content (OHC) in different ocean basins relative to corresponding control for the periodic 1× Pinatubo (named as PINA in the legend) and time-mean constant (named as CONST) volcanic perturbations in the intermediate ocean layers (300–1500 m). The x-axis here represents the time in years.

We also analyzed the post-eruption ocean heat content in different ocean basins and find that the decrease of the ocean heat content following explosive volcanism is relatively larger in the Atlantic and the Southern Ocean compared to the Pacific and the Indian Ocean (Fig. 8). This could be possible as the mean meridional overturning circulation caused by external radiative forcing in these two basins is stronger^45–47^. Moreover, the role of intermediate ocean layers (between 300–1500 m), especially of the Atlantic and the Southern Ocean basins, in heat adjustment (storage and redistribution) following strong volcanism is also larger compared to the upper and bottom ocean layers^46,47^. These results are consistent with earlier studies where a larger role of the oceanic intermediate layers in the heat uptake and adjustment, especially of the Atlantic and the Southern Ocean under increased global warming is discussed^48–50^. This larger role of intermediate ocean layers in setting the effective heat capacity is mainly related to the intensity of AMOC in these layers^51^.

### Atlantic ocean temperature

Observational and modeling studies have shown that the global oceans, in particular, the Atlantic Ocean, play a significant role in decadal climate variability^14,48–50^. The Atlantic Ocean responds vigorously to external radiative forcings, e.g., volcanism, due to the larger sensitivity of Atlantic Meridional Overturning Circulation to such forcings^45,51^.

**Figure 9:**
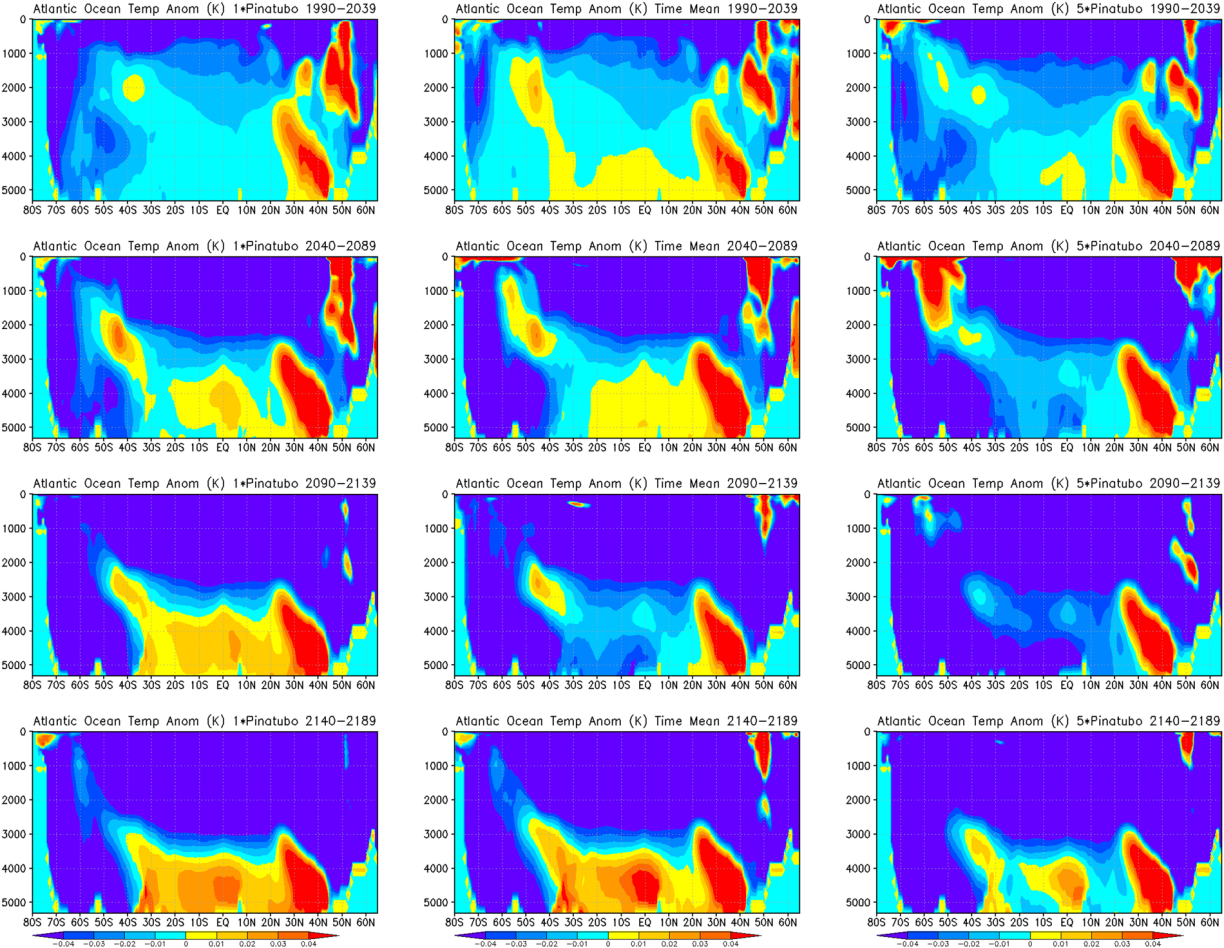
Zonal mean Ocean Temp Anomaly (K) displayed as a function of depth (y-axis, in meters) and latitude (x-axis) from the runs with periodic 1× Pinatubo/10a (left), time-mean volcanic aerosols (middle), and 5× Pinatubo/50a (right).

**Figure 10:**
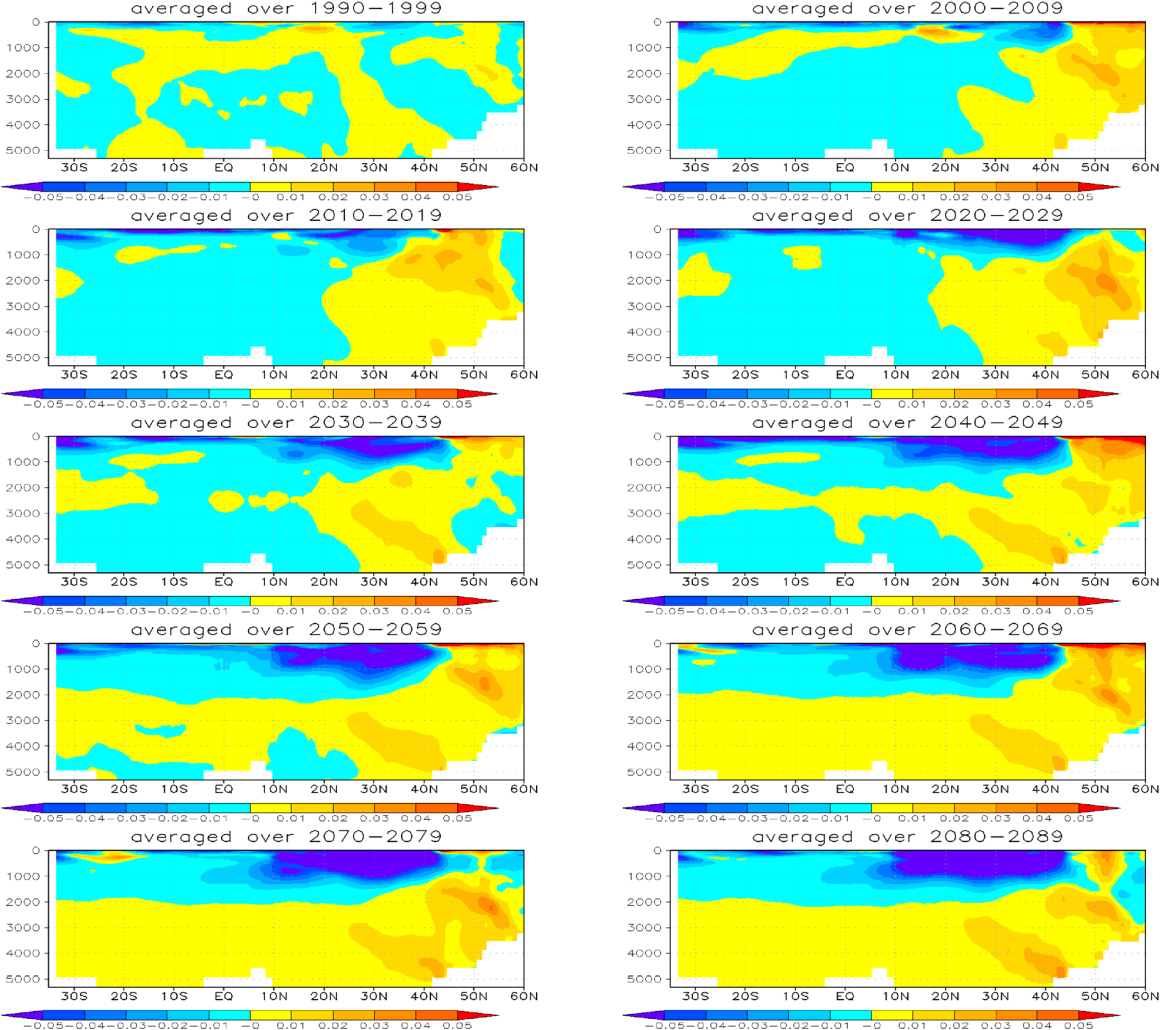
Zonal mean Atlantic Ocean salinity anomaly (PSU) from the runs with periodic 1× Pinatubo/10a.

**Figure 11:**
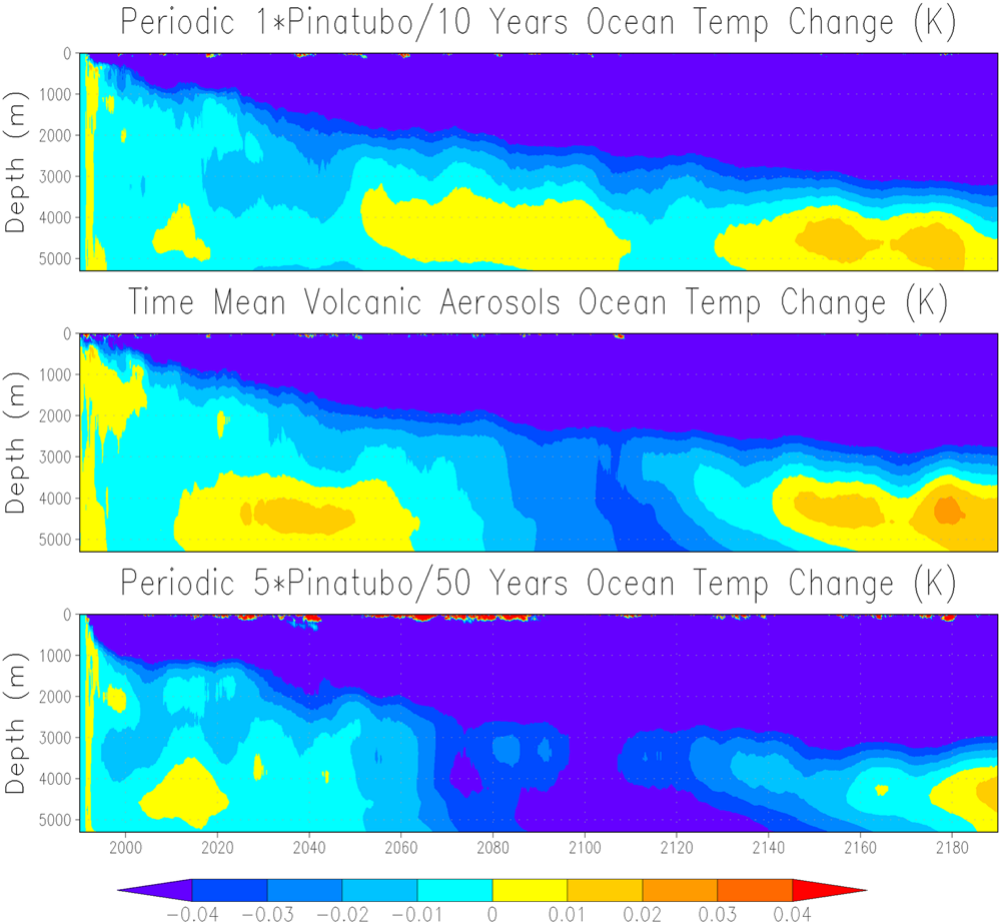
Ocean temperature anomaly (K) displayed as a function of depth and time from the runs with periodic 1× Pinatubo/10a (top), time-mean volcanic aerosols (middle), and 5× Pinatubo/50a (bottom).

**Figure 12:**
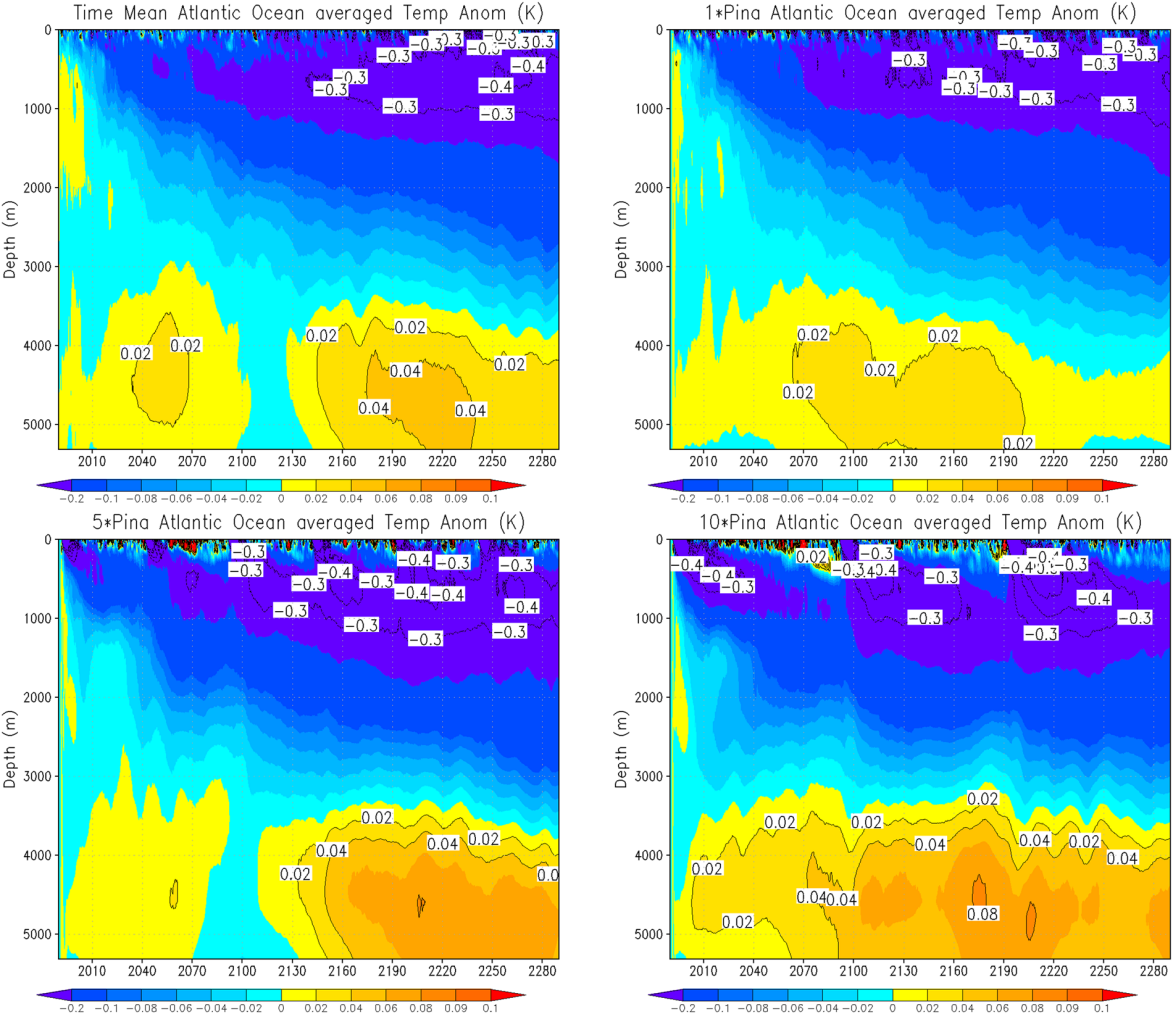
Atlantic Ocean ensemble-mean horizontally averaged ocean temperature anomaly as a function of depth and time for 1× Pinatubo/10a, constant forcing, 5× Pinatubo/50a and 10× Pinatubo/100a.

To get a better idea of post-eruption Atlantic Ocean temperature response, we show zonal mean 50-year average Atlantic Ocean temperature anomalies (for the first 200 years) of 1× Pinatubo, time-mean volcanic forcing, and 5× Pinatubo experiments (Fig. 9). The responses are fairly similar in all experiments; however, in 5× Pinatubo runs the cooling signal, especially in the deep ocean is slightly stronger. A warm anomaly is seen in all the cases at about 45–50°N, which penetrates downward and is accumulated at the bottom of the deep ocean. It is developed due to the sinking of warmer and denser saline water at high latitudes^14^. This warm anomaly in the deep Atlantic Ocean is a result of aerosol-induced cooling that strengthens the AMOC. The accumulation of this warm and saline water is clearly seen in Fig. 10, which shows the zonally averaged salinity profile for the first ten cycles of the periodic 1× Pinatubo/10a case. Figure 11 shows horizontally averaged Atlantic Ocean temperature anomaly as a function of depth and time for 1× Pinatubo, time-mean volcanic aerosol forcing and 5× Pinatubo experiments. The 5× Pinatubo experiment shows a slightly stronger cooling than other experiments (1× Pinatubo and time-mean experiments) indicating that the increase of cooling intensity tends to intensify downward mixing of cold water. Figure 12 shows the temporal evolution of Atlantic Ocean temperature anomalies for time-mean, 1×, 5× and 10× Pinatubo cases. We observed positive temperature anomalies in the upper ocean about 10 years after each eruption. It is better seen in 5× Pinatubo and 10× Pinatubo cases. These results are consistent with earlier studies, which also show a post-eruption ocean cooling and subsequent warming; especially, of the upper ocean^25,26,41^. This warming effect gets stronger with the increase of the amplitude of the radiative forcing. We notice that with the increase of the amplitude of volcanic forcing, a stronger surface warming is seen after each eruption cycle. This warming is a result of volcanic-induced surface cooling that produces a temperature gradient between the ocean surface and associated upper atmosphere that results in heat absorption and associated warming of the ocean surface following each eruption event^26^. Moreover, the warming in the lower ocean that eventually accumulates at the bottom is also increased and this effect gets stronger with the increase of the amplitude of the volcanic radiative forcing. This increased warming is caused as a result of the sinking of more saline and warmer water in the northern high latitudes (Figs. 9 and 10), as discussed by^14^. This air-sea temperature gradient mechanism operates in other ocean basins as well, however, in the Atlantic Ocean this volcanic-induced warmer, saline and denser water is penetrated down due to volcanic-induced strengthening of AMOC in the Atlantic Ocean compared to other Ocean basins, that is why Atlantic Ocean is important. Another important aspect is that the surface of the N. Atlantic is saltier (presumably due to less convection, more evaporation and more ice melting) than the surface of N. Pacific, making surface water denser in the N. Atlantic at the same temperature and leading to down-welling of water in this region, this difference is because on average N. Atlantic is warmer (10.0 °C) than N. Pacific (6.7 °C). This is mostly because of the greater local heating effect of the Gulf Stream, as compared to the Kuroshio Current. Warmer water evaporates more rapidly, creating a higher residual salt content.

**Figure 13:**
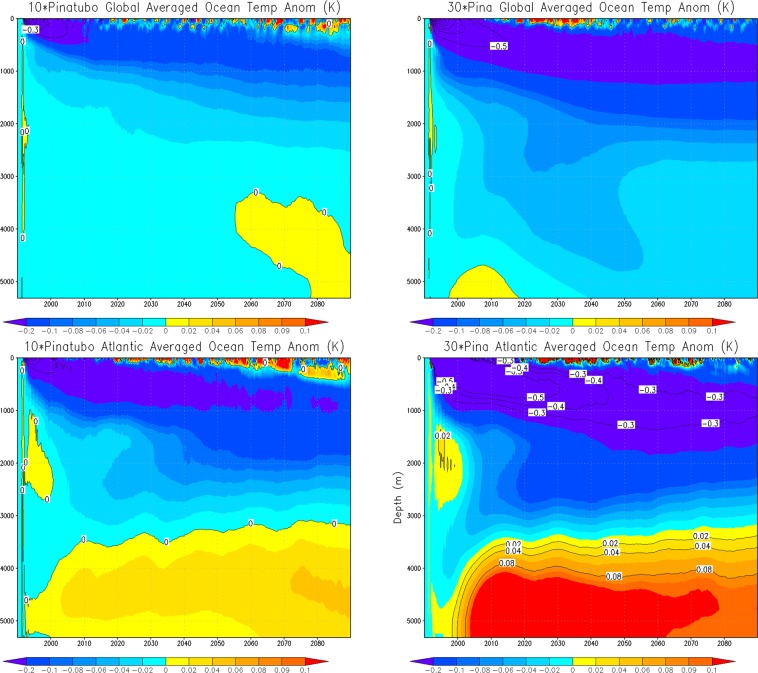
Ocean temperature anomaly (K) as a function of depth and time for global (top) and Atlantic Ocean (bottom) from the runs with periodic 10× Pinatubo/100a (left) and 30× Pinatubo/300a (right). Here the responses are shown for first 100 years.

To further understand the role of the mixed, thermocline and deep ocean layers, we extended our analysis and included another experiment that has much stronger volcanic forcing, i.e., 30× Pinatubo and compared it with 10× Pinatubo case (Fig. 13). The upper panel shows global averaged ocean temperature response and the lower panel shows the temperature response averaged over the Atlantic Ocean. The results reveal that the upper ocean mixed layer responds strongly to the increased external radiative forcing and shows much stronger surface warming following the massive eruption (except for some post-eruption years, that varies based on the amplitude of the eruption event). Moreover, the warmer, saline and dense water is also increased in the deep ocean layers as a result of increased overturning process following increased volcanic aerosol forcing. The cooling response in the thermocline and intermediate layers is also stronger for 30× Pinatubo case compared to 10× Pinatubo forcing. These results reveal that the lower (intermediate) ocean is playing a greater role in heat adjustment as it integrates and accumulates the cooling signal following each eruption. Enhanced warming in the ocean bottom layers (especially for the 30× Pinatubo case) suggests that increased periodic volcanic forcing results in stronger overturning especially in the Atlantic Ocean that allows the downward penetration and collection of more saline and dense water at the bottom.

**Figure 14:**
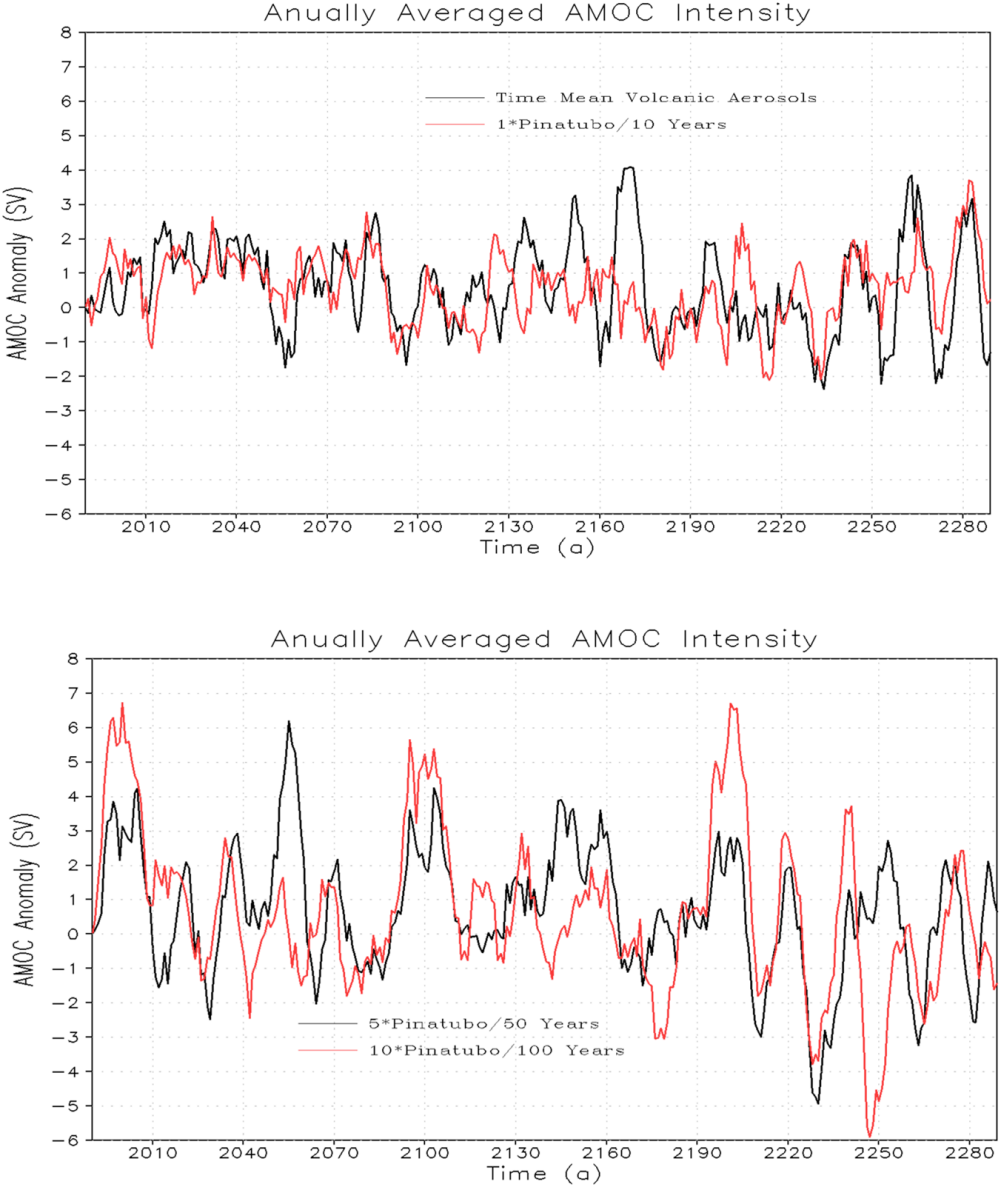
Annually averaged AMOC anomaly (Sv) for 1× Pinatubo/10a and constant forcing (top), 5× Pinatubo/50a and 10× Pinatubo/100a (bottom).

Earlier coupled climate model studies show that major tropical volcanic eruptions produce a significant intensification of the Atlantic Meridional Overturning Circulation^14^. Our analysis also confirms that Atlantic Meridional Overturning Circulation (AMOC) responds vigorously to volcanic impacts (Fig. 14). The AMOC anomaly does not accumulate but oscillates. Moreover, the response of AMOC for periodic and time-mean volcanic forcing is roughly similar despite there exist some small variations that could be accounted for by varying internal variability that need not be the same for both scenarios. The amplitude of the forced AMOC variability increases with the increase of the magnitude of the radiative forcing (Fig. 14, bottom panel).

### Ocean energy balance and AMOC

**Figure 15:**
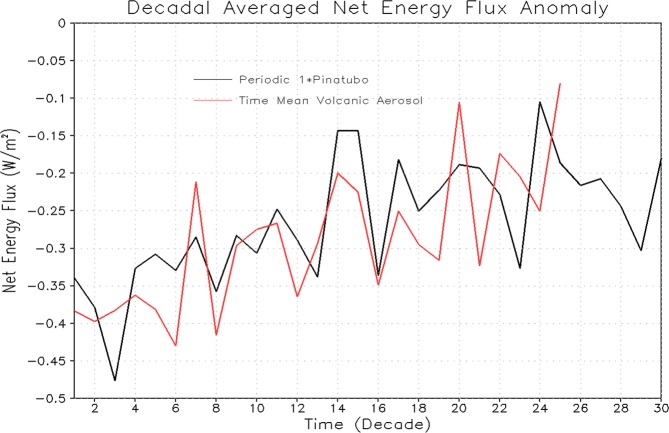
Decadal averaged Net Surface Flux anomaly (W/m^2^) averaged over the ocean surface from the runs with 1× Pinatubo/10a and time-mean aerosol forcing for the first 30 decadal cycles.

**Figure 16:**
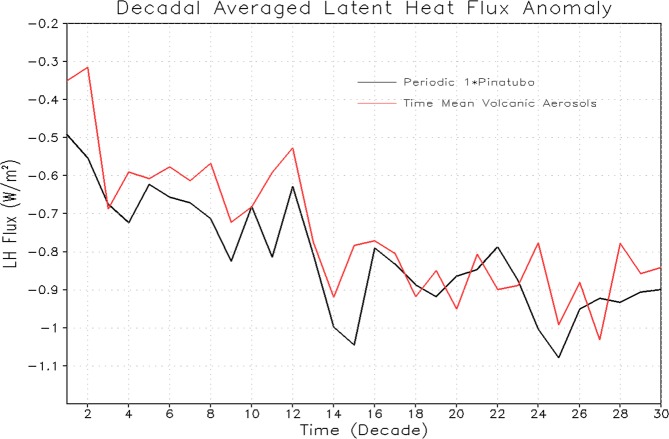
Decadal averaged Latent Heat (LH) Flux anomaly (W/m^2^) averaged over the ocean surface from the runs with 1× Pinatubo/10a and time-mean aerosol forcing for the first 30 decadal cycles. The negative LH flux anomaly i.e., decrease of LH flux with time leads to ocean warming.

The time scales of ocean response depends not only on the rate at which energy is absorbed (the net ocean heat uptake) at the ocean surface but also on the efficiency with which that energy is transported away from the surface and into the ocean interior^52^. The net energy flux at the ocean surface (computed as a decadal averaged anomaly for 1× Pinatubo and equivalent time-mean volcanic aerosols forcing experiments) decreases with time (Fig. 15). It indicates that the system equilibrates, as it absorbs heat (except for a few years after each large volcanic eruption event) from the atmosphere following each eruption, and warms the ocean surface (Fig. 6). The sensible heat flux following the explosive eruption event is positive suggesting that it cools the ocean (not shown). The latent heat flux anomaly is negative and tries to warm the ocean (Fig. 16). It happens because the latent heat is decreasing with time after the volcanic forcing, which means that the ocean surface is absorbing more heat and hence it will warm^14^. Downward net LW flux anomaly is also negative and it cools the system despite ocean temperature decreased (not shown). This is consistent with the results discussed in^14,21^.

**Figure 17:**
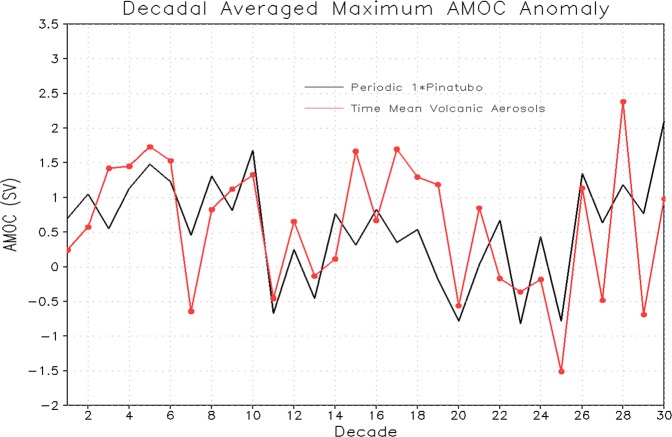
Decadal averaged maximum AMOC (Sv) anomaly from the runs with 1× Pinatubo/10a and time-mean aerosol forcing for the first 30 decadal cycles.

The rate of heat sequestration and transmission into the ocean interior is strongly correlated with the depth of heat penetration that appears to be regulated by the vertical extent and strength of the AMOC. We noticed that the AMOC response is highly variable and nonlinear. During the first couple of decades, there is a tendency of the AMOC strengthening but then it weakens (Fig. 17), suggesting that the response of AMOC is stronger following the initial few eruptions (periodic or time-mean) and then it slows down and system equilibrates. However, it strengthens again and shows multi-decadal variability with a periodicity of 40–50 years.

## Summary and Conclusions

The present study investigates the long-term oceanic response to explosive volcanism of constant and periodic nature of varying magnitude. It is anticipated that a sporadic strong cooling could initiate more vigorous vertical mixing of the upper ocean layer and therefore cools the ocean more effectively than a uniform radiative forcing. However, the long-term simulations show that on average the ocean heat content and sea-level rise responses to periodic and constant forcings are almost identical. To better understand this controversy, the oceanic response to periodic and energetically equivalent time-mean uniform volcanic eruptions of 1×, 5× 10× and 30× of Pinatubo size forcing is investigated with a focus on the North Atlantic Ocean, using a fully coupled atmosphere-ocean general circulation model (AOGCM), GFDL-CM2.1. The model is forced with a realistic volcanic radiative forcing of size 1×, 5× 10× and 30× of Pinatubo eruption, and is run for 300 years with 10, 50, 100 and 300 years repeating cycles respectively keeping all the other forcing factors fixed at 1860 level. The volcanic effects were evaluated using 3-member ensemble averaging that help to reduce the effect due to random noise.

We find that for a Pinatubo-size volcanic radiative forcing, the oceanic response to periodic eruptions and to the time-mean volcanic aerosols is close. This happens because of the comparable averaged AMOC response to the periodic and time-mean forcing. This is consistent with the results reported in earlier studies. However, the increase of the magnitude of the eruption could intensify “cold” uptake by the deep ocean. This tendency is observed for the 5× and 10× Pinatubo cases but is relatively weak. Our analysis further shows that ocean surface warms following each periodic eruption (except for few post-eruption years) and this warming of the surface is stronger for stronger (e.g., 5×, 10× and 30×) eruptions. It follows Newton’s law of cooling and heating. This post-eruption ocean surface warming allows the upper ocean to equilibrate, however, deep ocean layers integrate the cooling signal and play an important role in ocean heat adjustment. A warm anomaly in the Atlantic Ocean is seen at about 45°N to 50°N. It penetrates downward and is accumulated in the deep ocean layers. It is developed due to the sinking of warmer and denser saline water at high latitudes. The warming of both upper surface and bottom layer intensifies with increasing of the amplitude of forcing leading to weakening of ocean stratification. The amplitude of forced AMOC variability increases with the amplitude of radiative forcing, however, the increase is not continuous. We currently conduct experiments with 100× Pinatubo forcing to better quantify the magnitude threshold and to see if the periodic and time-mean volcanic forcing could initiate different responses with the increase of the strength of volcanism.

Our results further reveal that the ocean heat content decrease following explosive volcanism is relatively larger in the Atlantic and the Southern Ocean compared to the Pacific and the Indian Ocean. This happens because the mean meridional overturning circulation following a series of strong volcanism in these two basins is stronger than in the Pacific and the Indian Ocean. Moreover, the role of intermediate ocean layers, especially of the Atlantic and the Southern Ocean, in heat adjustment, heat storage and redistribution following strong volcanism is larger compared to the upper and bottom ocean layers. This larger role of intermediate ocean layers in setting the effective heat capacity is mainly related to the intensity of AMOC in these layers. These findings are important for better understanding of the post-eruption ocean heat uptake, ocean heat adjustment, redistribution, ocean relaxation mechanism and for geoengineering applications in long-term climate studies.
